# Successfully Engaging Private Providers to Improve Diagnosis, Notification, and Treatment of TB and Drug-Resistant TB: The EQUIP Public-Private Model in Chennai, India

**DOI:** 10.9745/GHSP-D-18-00318

**Published:** 2019-03-22

**Authors:** Ramya Ananthakrishnan, M. D'Arcy Richardson, Susan van den Hof, Radha Rangaswamy, Rajeswaran Thiagesan, Sheela Auguesteen, Netty Kamp

**Affiliations:** a REACH (Resource Group for Education and Advocacy for Community Health), Chennai, Tamil Nadu, India.; b KNCV Tuberculosis Foundation, The Hague, Netherlands. Now an independent consultant, San Francisco, CA, USA.; c KNCV Tuberculosis Foundation, The Hague, Netherlands. Now with the National Institute of Public Health and the Environment (RIVM), Bilthoven, The Netherlands.; d KNCV Tuberculosis Foundation, The Hague, Netherlands.

## Abstract

**Background:**

Private physicians in India see and treat more than half of all people with tuberculosis (TB) each year and thus have potential to make significant contributions to TB control. The EQUIP project was designed as a prospective cohort study to assess the potential of private providers to diagnose and appropriately treat drug-resistant TB (DR-TB) in the Central and South districts of Chennai, India.

**Methods:**

The private-sector engagement model consisted of free access to rapid diagnostics; choice of free daily or thrice-weekly treatment regimens; support for notification of patients; and patient support including directly observed therapy through EQUIP centers staffed by a community-based interface agency. Data were collected on provider participation; referral results; treatment regimens prescribed; and treatment outcomes.

**Results:**

From October 2015 through June 2017, 227 of the 466 (48.7%) private providers approached referred at least 1 patient to an EQUIP center for evaluation. A total of 2,621 patients received testing and 1,232 (47.0%) were diagnosed with TB. Of those, 727 (59.0%) were bacteriologically confirmed, including 694 (56.3%) using GeneXpert and 33 (2.7%) using smear microscopy. A total of 26 (3.7% of GeneXpert diagnosed) patients were confirmed as rifampicin-resistant cases. EQUIP-related notifications comprised approximately 10% of TB and DR-TB notifications in Chennai during the project period. The project initiated 1,167 (96.8%) drug-sensitive TB patients on treatment. Of those, 691 (59.2%) received standard daily regimens with EQUIP support and 288 (24.7%) received standard intermittent regimens. At the time of writing, 89.4% of 868 drug-susceptible TB patients receiving EQUIP support had treatment success. Of the 26 rifampicin-resistant TB cases notified, 20 (77%) started and continued on second-line treatment; 2 died and 4 were lost to follow-up prior to treatment initiation.

**Conclusion:**

Private providers can make a substantial contribution to detection and appropriate treatment of patients with TB and DR-TB in India when provided with access to rapid diagnostics, support for notification and patient treatment through interface agencies, and free, quality anti-TB drugs.

## Abstract (precis)

Based on a participatory program design that addressed the self-described needs of private providers, a local NGO offered the providers access to rapid diagnostics and support for notification and patient treatment including free anti-TB drugs. The model resulted in high provider participation, contributing more than 10% of the overall TB case notifications, and an 89% treatment success rate for drug-sensitive TB.

## INTRODUCTION

India has the largest burden of tuberculosis (TB) and drug-resistant tuberculosis (DR-TB) in the world, with an estimated 2.8 million new cases of TB occurring annually, of which 5% are DR-TB requiring second-line treatment.^1^

In Chennai, the capital of Tamil Nadu state, an estimated 15,185 TB cases occur each year but only about 8,600 are notified, leaving more than half of the incident TB cases unaccounted for.^2^^,^^3^ One important factor is the large, diverse, and poorly coordinated private health sector, where more than 60% of people with TB first seek care.^4^ Based on available evidence,^5^^–^^11^ there is continuing concern about lack of notification of privately diagnosed TB cases, inappropriate diagnostic confirmation of these cases, inappropriate treatment regimens, use of low-quality drugs, and lack of support for treatment completion among private-sector patients.

Many efforts have been launched to engage the private sector effectively in TB control, with varying degrees of success.^12^^–^^15^ Since 1998, the Resource Group for Education and Advocacy for Community Health (REACH) has worked to increase patient access to the public health services of India's Revised National TB Control Program (RNTCP). REACH encourages private health care providers to refer their patients to one of REACH's 4 public-private mix centers in Chennai, where REACH ensures continuity of care between the private and public sectors through project-implemented counseling, education, food support, and directly observed treatment (DOT).^16^ Using lessons learned from this work and from focus group discussions with private providers and patients, REACH and KNCV Tuberculosis Foundation developed and tested a model under Project EQUIP (Enhanced Use of Quality Drugs and Utilization of Innovative Diagnostics for TB Management in the Private Sector) to evaluate the potential for private providers to contribute to appropriate diagnosis and treatment of DR-TB.

Many efforts have been launched to engage the private sector effectively in TB control, with varying degrees of success.

At the point of project launch, no other effort had focused specifically on the issue of private providers' engagement in prevention, diagnosis, and treatment of DR-TB. EQUIP set out to demonstrate a sustainable model for private-sector engagement in DR-TB; encourage private providers to use state-of-the-art diagnostics for their patients with TB symptoms; promote the use of standardized TB and DR-TB treatment regimens with quality-assured drugs; and provide coordinated support for private-sector patients to improve treatment success. While data were collected, analyzed, and are presented here for both TB and DR-TB, the primary question of interest was what effect private-sector engagement would have on notification and treatment of DR-TB in Chennai.

The project operated between April 2015 and June 2017, with patient follow-up until December 2017, in 2 of the 3 districts of Chennai (Central and South), comprising a population of approximately 5.3 million people.

## METHODS

### Study Design

The study used a prospective cohort design. The primary cohort of interest was the group of private providers in the Central and South districts of Chennai who were oriented to the project and who agreed to participate. The secondary cohort of interest was the group of private-sector patients who were diagnosed by participating private providers between October 1, 2015 and June 30, 2017.

**Figure:**
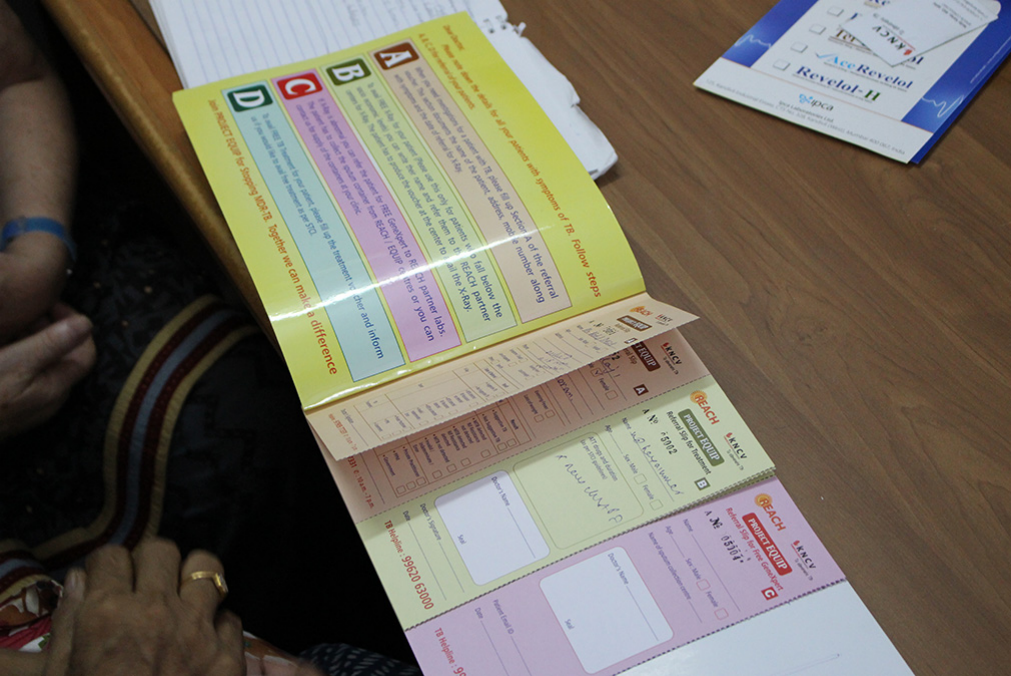
Simple guidance steps and vouchers provided by the project allowed private providers to easily refer patients for TB diagnostic testing. © Jasper Hamann

### Setting

Chennai is a metropolitan city in Tamil Nadu with a total estimated population of 7,196,515. The public-sector TB control program in Chennai has been implemented by the Greater Chennai Corporation. Greater Chennai Corporation covers 15 zones across 36 TB units and is subdivided into 3 districts—North, Central, and South Chennai. South Chennai has a historically lower case detection rate than North and Central Chennai. This project was implemented in Central and South Chennai with the population of 5,387,132 covering 27 TB units.

### Formative Research

**FIGURE 1:**
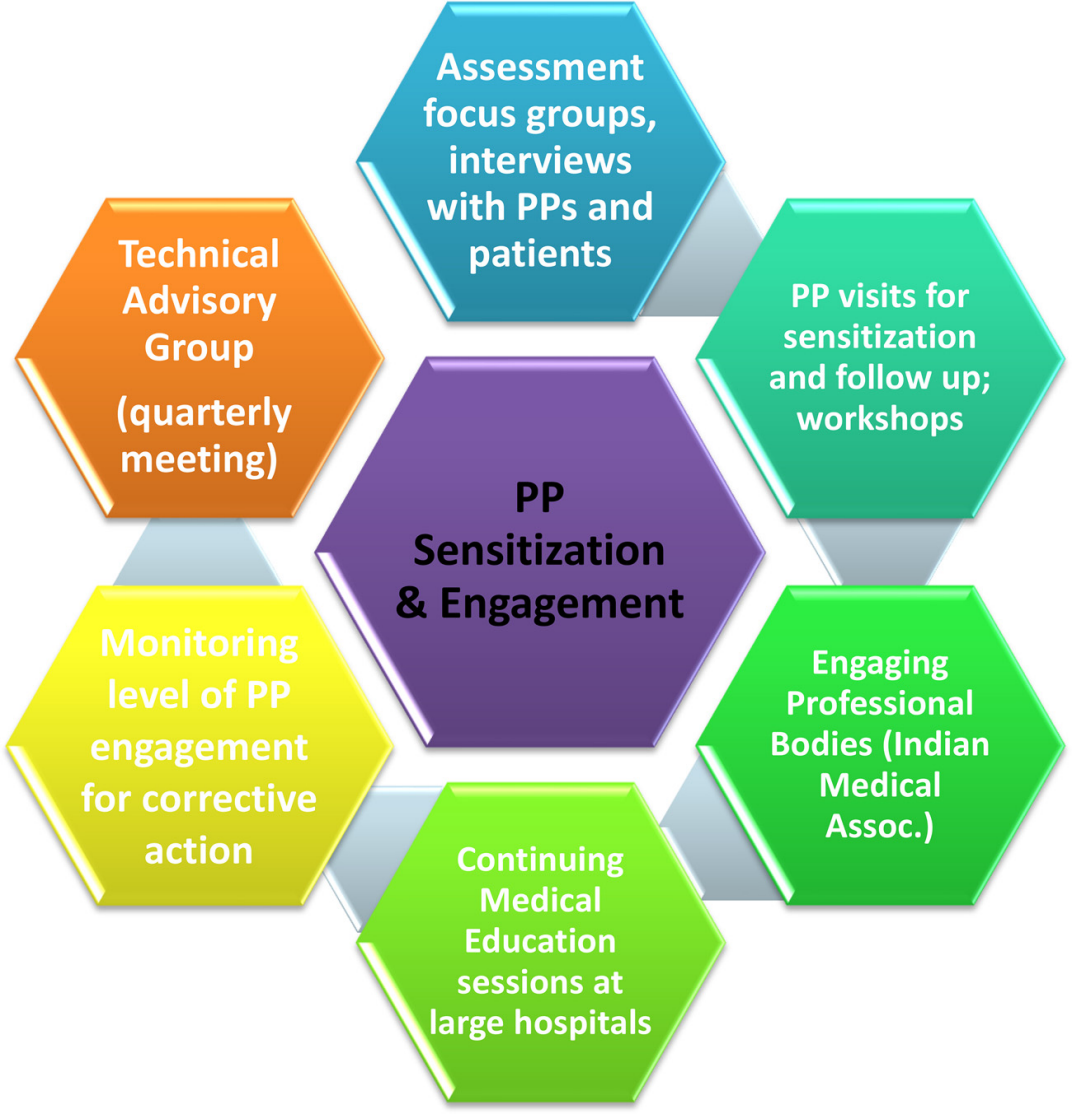
The Private Provider Engagement Process Under Project EQUIP Abbreviations: EQUIP, Enhanced Use of Quality Drugs and Utilization of Innovative Diagnostics for TB Management in the Private Sector; PP, private provider.

REACH and KNCV designed the EQUIP model with the hypothesis that involving the target audience (private providers and their patients) at the beginning of the process would lead to a high participation rate by directly addressing their self-identified needs. To do so, we mapped the private provider landscape and selected a subgroup of providers—chest physicians, general practitioners, and selected specialists—who likely saw high numbers of people with symptoms of TB. We then convened focus group discussions and individual interviews with private provider and patient representatives to understand barriers to engagement in public TB control efforts. In addition, we formed an advisory group of well-respected senior private practitioners—chest physicians, microbiologists, pediatricians, and TB experts from the National Institute for Research in Tuberculosis; representatives from the Indian Medical Association; and the District TB Officer and Superintendent of the Tambaram Sanatorium (the tertiary referral hospital)—to provide advice on the model. The advisory group continued to meet after the initial phase during quarterly meetings organized by REACH to update members on progress and seek technical expertise and guidance. Suggestions from the group were debated and incorporated into the model over the course of the project. The engagement process is depicted in Figure 1.

### Data Collection and Analysis

A Microsoft Excel database already in use at REACH to document care for TB patients diagnosed and treated with support from REACH was expanded to record information on individual provider characteristics and behaviors, including type of provider (general practitioner, chest physician, or specialist), number of referrals for TB testing over time, and number of patients diagnosed with TB. Data regarding the basic sociodemographic, diagnostic, and source of referral details were collected and recorded for all patients referred who reached the diagnostic step of the pathway. For all diagnosed TB patients, basic clinical, sociodemographic, and treatment regimen and outcome details from the standard treatment card were entered into the database. The data were compared for consistency and all inconsistencies were resolved by referring to the treatment card. Data were analyzed using IBM SPSS Statistics 20 for Windows 8. Univariate analysis was conducted and the results were expressed in proportions. We used standard World Health Organization definitions related to TB diagnosis and treatment outcomes for purpose of the study.^17^ Only the key project personnel and data manager had access to the project data, which were stored in a password-protected non-networked computer.

### Ethical Approval

The study design was reviewed and approved by the Independent Ethics Committee of REACH.

## RESULTS

### Formative Research Findings From Consultations With Private Providers

Focus group discussions and individual interviews with our target groups of providers yielded the following key information used in developing our engagement model.

**Many private physicians preferred daily treatment over the thrice-weekly treatment regimen** that was free and supported by the RNTCP and national guidance documents. Reasons for this were twofold: (1) they did not believe the thrice-weekly treatment regimen was adequate treatment and therefore thought it may result in more relapses, and (2) they believed the higher doses required in the thrice-weekly regimen led to increased side effects and higher discontinuation of treatment for patients. Since the thrice-weekly regimen was the only one available through RNTCP at the time of project implementation, many providers preferred not to cooperate with the RNTCP but to prescribe and treat independently using a daily regimen that patients had to purchase. The RNTCP supported the daily regimen and planned to start it in the country in pilot districts at the time the project started.

Many private physicians preferred a daily TB treatment regimen over the thrice-weekly regimen.

**Most private providers were unaware of the new diagnostic technology available** for TB (GeneXpert) or were unsure of its reliability. After learning more about it during the orientation sessions, most expressed interest in accessing the technology to improve the accuracy and turnaround time between testing and result. They saw this as a way to improve customer service and satisfaction.

**Private providers perceived the TB case notification process as too time-consuming**. Many were unfamiliar with the national online notification platform (Nikshay) available since 2013 and did not have direct access to report cases themselves. In addition, providers were concerned about patient confidentiality, especially for their more affluent patients who might lose social standing in their communities if discovered to have TB.

**The vast majority of private providers preferred not to treat DR-TB patients**, for several reasons: (1) the higher likelihood of a poor outcome and therefore damage to the provider's reputation; (2) perceived increased risk of infection to themselves and their staff; (3) lack of second-line drugs in pharmacies; and (4) lack of skills and experience in treating complex DR-TB cases. Almost all providers preferred to refer these patients to RNTCP facilities for second-line treatment. However, DR-TB was usually only diagnosed after the initial treatment prescribed by the private provider had failed to cure the patient.

Focus group discussions and individual interviews with patients revealed that TB had important financial implications for them, due to costs for diagnosis and treatment as well as reduced income. In addition, TB continues to be surrounded by stigma, leading patients to be unwilling to disclose their status to others or allow home visits by DOT supporters.

**FIGURE 2:**
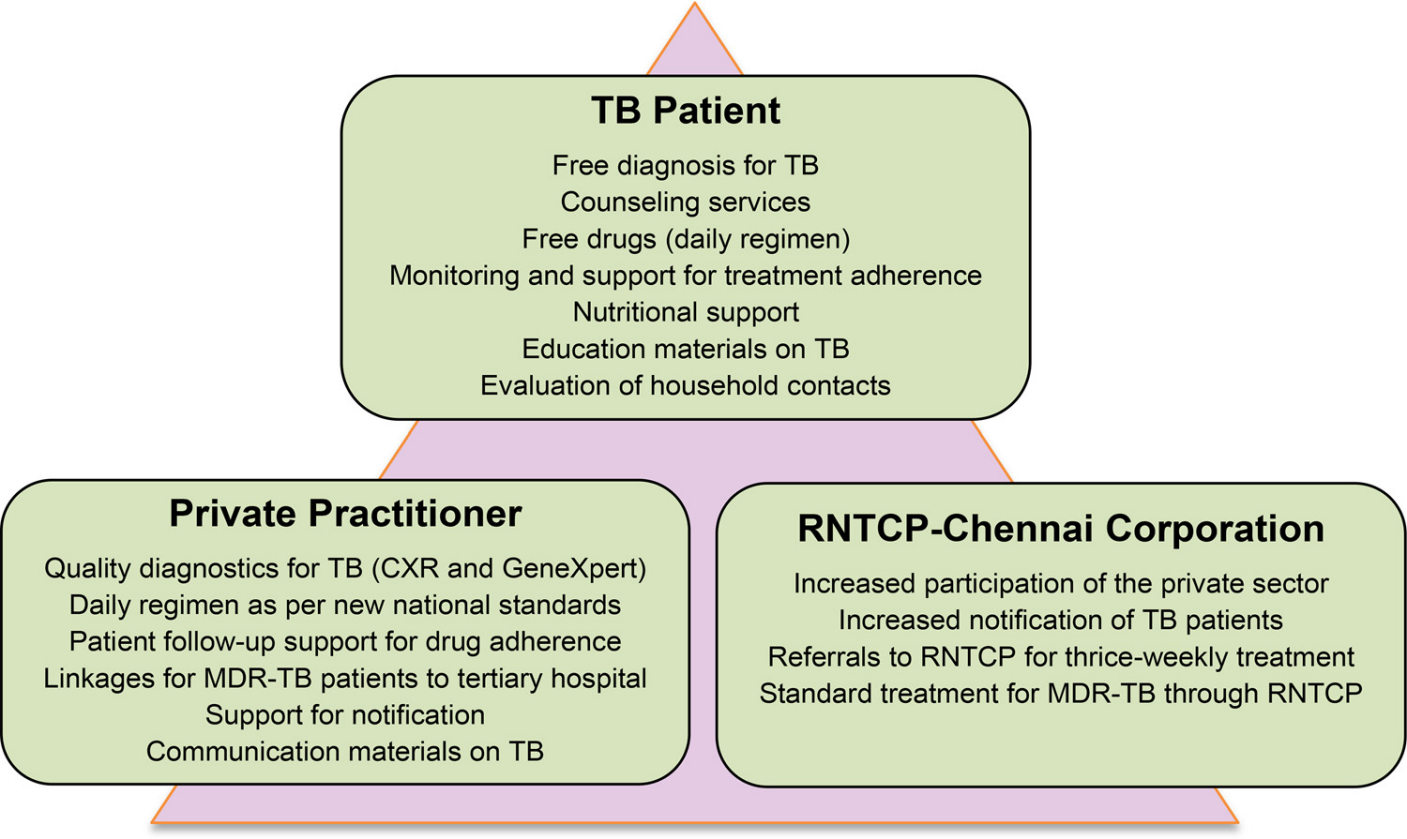
Benefits of the EQUIP Model for Participating Groups Abbreviations: CXR, chest x-ray; EQUIP, Enhanced Use of Quality Drugs and Utilization of Innovative Diagnostics for TB Management in the Private Sector; MDR-TB, multidrug-resistant tuberculosis; RNTCP, Revised National TB Control Program; TB, tuberculosis.

**FIGURE 3:**
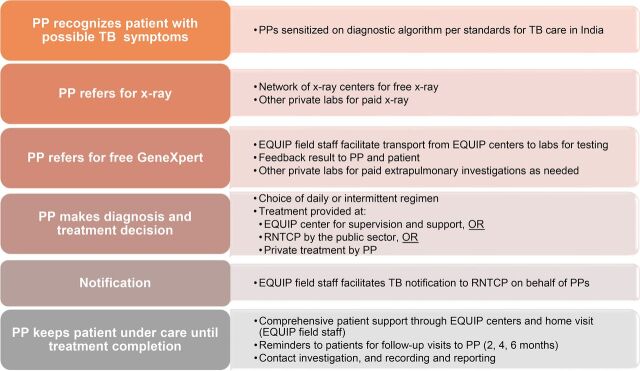
Private-Sector TB Patient Pathway to Cure in the EQUIP Model Abbreviations: EQUIP, Enhanced Use of Quality Drugs and Utilization of Innovative Diagnostics for TB Management in the Private Sector; PP, private provider; RNTCP, Revised National TB Control Program; TB, tuberculosis.

The EQUIP model was designed to address these concerns. The key components of the model are the provider engagement modality through one-to-one visits; the continuous regular involvement of a technical group of public and private technical experts; the access to free GeneXpert testing and preferred daily TB drugs; the choice for the provider and patient to decide whether the patient will receive treatment at the EQUIP centers at private facilities, another private practice, or referral to the RNTCP public centers; and the EQUIP field staff support to both patient and provider during the entire cascade of TB care. Box 1 summarizes these key features, and Figure 2 presents the benefits for each group of participants in the project. Figure 3 depicts the private-sector patient pathway from presentation to diagnosis and treatment completion. Of note, unlike a number of other private-sector engagement models, private providers received no direct or indirect financial compensation for participating in the EQUIP network.

BOX 1 **Key Features of the EQUIP Model**

- Providers were recruited to participate through an initial sensitization visit and orientation to standards of TB care in India, including diagnosis and treatment per the national guidelines.
- EQUIP staff conducted monthly one-on-one follow-up visits with providers to actively involve and maintain their interest in diagnosing and treating TB patients while reducing their effort and time investment to come to meetings.
- All participating providers received access to free diagnostics with chest x-ray and GeneXpert through a voucher system.
- EQUIP centers, located at private health facilities and staffed by the EQUIP project, provided a free interface between private providers and patients.
- Choice of the treatment regimen (thrice-weekly or daily) and whether to receive DOT at the EQUIP centers, with support of community volunteers, or by the private doctor was decided by the private provider and patient.
- EQUIP field staff:
  - Instructed referred patients how to produce a sputum specimen and where to go for testing
  - Transported specimen to GeneXpert sites as needed
  - Provided rapid reporting of results from chest x-ray and GeneXpert facilities to referring doctor by email and/or SMS
  - Assisted private providers with the TB case notification process
  - Provided access to free quality-assured drugs for TB treatment using either a thrice-weekly or a daily treatment regimen (supplied by RNTCP or through EQUIP-funded pharmacy vouchers, respectively)
  - Facilitated quick referral for diagnosed DR-TB patients for treatment initiation at the public referral hospital
  - Offered patient and private provider-friendly communication materials
  - Provided counseling services for treatment adherence and mitigation of the social impact of TB
  - Offered conditional nutritional enablers for TB patients through a coupon system
  - Gave ongoing feedback to private providers on patient status

### Engagement of Providers

A total of 466 private practitioners were approached and oriented during 7 quarters of project activity. Of those, 12% were chest physicians, 65% general practitioners, and 23% specialists (pediatricians, gynecologists, and orthopedic specialists). Of the 466 providers sensitized, 227 (48.7%) actively participated during the project period by referring 1 or more patients with TB symptoms for diagnosis and/or treatment support through EQUIP. After initial orientation visits, providers were encouraged to refer their patients through monthly one-on-one visits. Most of the active private providers referred a patient within 1 to 3 months of agreeing to participate in the EQUIP network. While participation in the project was high, a much smaller subset of providers (the “super-referrers”—those referring 30 or more patients during the project) accounted for a high proportion of the patients referred. Twenty-one providers (9.3% of all active providers) accounted for approximately 48% of total referrals, while more than half of the engaged providers referred only 1 to 5 patients over the life of the project.

Of the 466 providers sensitized to the private-sector engagement model, nearly 50% actively participated in it.

As is often the case, there were a few “super-referrers” among the practitioners who participated, who accounted for the majority of the referrals. Specificially, 21 providers (9.3% of all active providers) accounted for approximately 48% of total referrals, while 142 (62.6%) providers referred only 1 to 5 cases over the life of the project (Table 1). In discussions with the providers, they offered 2 explanations for the super-referrer phenomenon: (1) chest physicians receive a number of referrals from general practitioners for suspected cases of TB and therefore have a concentrated high-risk patient load, and (2) certain providers are situated close to high-burden areas such as slums or low-income population centers.

**TABLE 1.** Number of TB Referrals by Individual Providers

No. of Referrals	No. (%) of Providers	CumulativePercentage
1–5	142 (62.6)	62.6
6–10	33 (14.5)	77.1
11–30	31 (13.7)	90.7
31–50	14 (6.2)	96.9
51–70	2 (0.9)	97.8
71–100	2 (0.9)	98.7
>100	3 (1.3)	100.0
**Total**	**227 (100.0)**	

It appears that all types of providers contribute substantially to identifying TB cases, particularly general practitioners because of their sheer numbers (Table 2). Chest physicians had the highest yield, at 53.6% of their referred cases, while general practitioners and specialty physicians yielded 44.9% and 41.4% of cases, respectively. There are clearly a small number of highly active participants in TB case identification. Table 3 details the TB referrals among super-referrers only. For chest physicians and general practitioners, the yields of TB cases from their referrals (53.3% and 41.5%, respectively) are similar to the overall respective population of providers (53.6% and 44.9%, respectively), but they also account for a very large proportion of the cases notified. The 8 high-referring chest physicians represented only 20% of the participating chest physicians, but accounted for 61% of the patients referred by chest physicians and 61% of the TB cases diagnosed. High-referring general practitioners accounted for 7.7% of the participating general practitioners but contributed 45% of the referrals and 41.5% of the TB cases identified by general practitioners. In contrast, 2 high-referral specialists (4.5% of participating specialists) had a much lower yield than the overall population of their colleagues, at 22.2% versus 41.4% for all specialists. However, these 2 specialists accounted for 31.6% of referrals and 17% of TB cases diagnosed through specialists. These data are more difficult to interpret due to the inherent difficulties in diagnosing extra-pulmonary TB; recommendations on engaging specialists would depend on additional investigation and analysis.

**TABLE 2.** Number and Yield of TB Referrals by Type of Provider and Quarter

Quarter and Year	Chest Physician			General Practitioner			Specialty Physician			Total		
	No. of Referrals	No. of TB Cases	Yield (%)	No. of Referrals	No. of TB Cases	Yield (%)	No. of Referrals	No. of TB Cases	Yield (%)	No. of Referrals	No. of TB Cases	Yield (%)
Q4 2015	40	28	70.0	46	39	84.8	8	6	75.0	94	73	77.7
Q1 2016	81	43	53.1	154	101	65.6	32	19	59.4	267	163	61.0
Q2 2016	101	60	59.4	128	69	53.9	30	15	50.0	259	144	55.6
Q3 2016	110	61	55.5	207	108	52.2	25	9	36.0	342	178	52.0
Q4 2016	109	47	43.1	233	104	44.6	48	18	37.5	390	169	43.3
Q1 2017	153	82	53.6	380	139	36.6	65	17	26.2	598	238	39.8
Q2 2017	151	78	51.7	443	155	35.0	77	34	44.2	671	267	39.8
**Total**	**745**	**399**	**53.6**	**1591**	**715**	**44.9**	**285**	**118**	**41.4**	**2621**	**1232**	**47.0**

Abbreviations: Q, quarter; TB, tuberculosis.

**TABLE 3.** Number and Yield of TB Referrals Among Super-Referrers Only (>30 Referrals), by Type of Provider

Type of Provider	No. of Referrals	No. of TB Cases	Yield (%)
Chest physician (n=8)	458	244	53.3
General practitioner (n=11)	716	297	41.5
Specialty physician (n=2)	90	20	22.2
**Total (N=21)**	**1264**	**561**	**44.4**

Abbreviation: TB, tuberculosis.

### Referral, Diagnosis, and Notification

**FIGURE 4:**
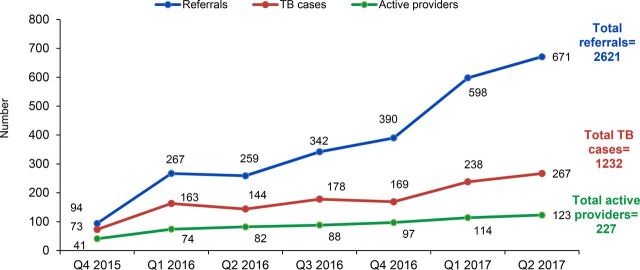
Private Provider Engagement, Referrals for TB Diagnosis, and TB Cases, October 2015–June 2017

The 227 active providers referred a total of 2,621 patients for diagnostic tests, by chest x-ray, and/or GeneXpert (Figure 4). Of the 2621 patients referred by private providers for TB diagnosis, 1,232 (47.0%) were diagnosed with TB, of which 727 (59.0%) were bacteriologically confirmed (Table 4), including 694 (56.3%) using GeneXpert and 33 (2.7%) with positive sputum smear microscopy but negative GeneXpert. In addition, 265 (21.5%) patients were diagnosed by abnormal chest x-ray (a common diagnostic of choice in the private sector) and the remaining 240 (19%) were diagnosed based on other laboratory testing (e.g., magnetic resonance imaging [MRI], fine needle aspiration cytology [FNAC], biopsy, or histopathology) or clinical suspicion only. This compares favorably with overall diagnostic practices reported by the RNTCP for these 2 districts in Chennai: in 2016, 55% of all notified cases in Central Chennai and 54% in South Chennai were bacteriologically confirmed.

Of the 2,621 patients referred by private providers for TB diagnostic tests, 47% were diagnosed with TB.

**TABLE 4.** Number of Patients Referred to EQUIP by Type of Private Provider

	Provider Type			Total (n=227)
	Chest Physicians (n=40)	General Practitioners (n=143)	Specialists (n=44)	
No. of referred patients	742	1,592	287	2,621
No. of patients referred for GeneXpert testing	620	1,158	167	1,945
No. of patients diagnosed with TB	396	716	120	1,232
No. (%) of patients diagnosed with TB with bacteriological confirmation (GeneXpert/SSM)	243 (61.3%)	436 (60.8%)	48 (40.0%)	727 (59.0%)
No. (%) of patients confirmed with RR-TB	NA	NA	NA	26 (3.7%)

Abbreviations: EQUIP, Enhanced Use of Quality Drugs and Utilization of Innovative Diagnostics for TB Management in the Private Sector; RR-TB, rifampicin-resistant tuberculosis; SSM, sputum smear microscopy; TB, tuberculosis.

Among the 694 specimens positive for TB with GeneXpert, 31 tested positive for rifampicin resistance. Five of those were later determined to be drug-sensitive using conventional drug susceptibility testing (Line Probe Assay or Mycobacteria Growth Indicator Tube) or repeat GeneXpert, for a total of 26 (3.7%) confirmed rifampicin-resistant cases found through GeneXpert testing and notified to RNTCP during the project period.

All 1,232 patients diagnosed through EQUIP were notified to the RNTCP. In addition, private providers also requested EQUIP to notify 36 patients they had diagnosed and managed themselves outside of the project. By comparison, in previous years the private sector accounted for substantially fewer notifications: 301 in 2013, 487 in 2014, and 524 in 2015 in all 3 districts of Chennai.

### Treatment Regimens and Outcomes for TB and DR-TB

Of the 1,206 patients diagnosed without rifampicin resistance, 1,167 (96.8%) initiated treatment, 11 (0.9%) died, 13 (1.1%) were lost to follow-up, and another 13 (1.1%) transferred out prior to treatment start. Among those who started treatment, 691 (59.2%) received standardized daily regimens with treatment support provided by EQUIP. In addition, 288 (24.7%) received standardized intermittent regimens, either delivered by EQUIP (n=177) or through public RNTCP facilities (n=111), while 185 (15.9%) bought private prescriptions on their own. Only 3 patients refused to be treated with standard allopathic anti-TB regimens and instead chose to visit traditional practitioners who prescribed traditional (herbal or ayurvedic) medicines.

At the time of writing, 89.4% of the 868 patients with drug-susceptible TB who received treatment through EQUIP and were eligible to have completed their treatment by this date had done so successfully (Table 5). Of the 26 rifampicin-resistant TB cases notified, 20 (77%) were started on second-line treatment. Nineteen of those started treatment through the RNTCP system and one started treatment through a private provider. Two patients treated through RNTCP died shortly after treatment initation. Of the 6 patients who did not start treatment, 2 died and 4 were lost to follow-up prior to treatment initiation.

89% of patients with drug-susceptible TB who received treatment by the project had treatment success.

**TABLE 5.** Treatment Outcomes for Patients Without Confirmed RR-TB and Who Received Treatment Support Through EQUIP

Treatment Outcomes	Patients Referred by:			Total (n=868)
	Chest Physicians (n=226)	General Practitioners (n=513)	Specialist Physicians (n=129)	
Completed, No.	182	350	108	640
Cured, No.	21	103	12	136
Treatment success, No. (%)	203 (89.8%)	453 (88.3%)	120 (93.0%)	776 (89.4%)
Died, No.	9	20	5	34
Lost to follow-up, No.	13	29	4	46
Transferred to private facility treatment, No.	0	2	0	2
Transferred to RNTCP facility treatment, No.	1	5	0	6
Treatment failure, No.	0	3	0	3
Still on treatment, No.	0	1	0	1

Abbreviations: EQUIP, Enhanced Use of Quality Drugs and Utilization of Innovative Diagnostics for TB Management in the Private Sector; RNTCP, Revised National TB Control Program; RR-TB, rifampicin-resistant tuberculosis.

## DISCUSSION

Many efforts have been launched to engage the private sector effectively in TB control in India. A number of those models have been successful in increasing case notifications but have been difficult to expand because priority has been given to strengthening the public sector with less emphasis on creating lasting partnerships with private-sector providers. The EQUIP model shares a number of characteristics with other models, including the Strengthening Health Outcomes through the Private Sector (SHOPS) project, supported by the United States Agency for International Development (USAID) and implemented by Abt Associates from 2012 through 2015, on which the successful Mumbai Public-Private Interface Agency (PPIA) project was later modeled. All of these models mapped, recruited, and trained local private providers; provided support for notification; increased access to diagnostic technologies; and provided standardized treatment as well as treatment support to patients through an interface agency. There are several differences in EQUIP to note. First, the EQUIP model is the only effort to have focused specifically on DR-TB detection in the private sector. Second, EQUIP actively engaged the target population of providers in formative research and project design and continued to consult with them on an ongoing basis. Third, EQUIP's centers were established within existing private-sector facilities and relied more heavily on in-person support to help patients navigate the complex health care system, as opposed to electronic communication. Fourth, EQUIP sought to establish diagnostic sites within their own network of private facilities to increase the convenience for providers and patients. Fifth, the EQUIP model was the only one to offer a daily treatment option to providers and patients. Finally, in an effort to increase chances for sustainability, the model offered no direct or indirect compensation to private providers for participating in the network, unlike the other models that have included cash transfers, phone minutes, or other rewards.

**Figure:**
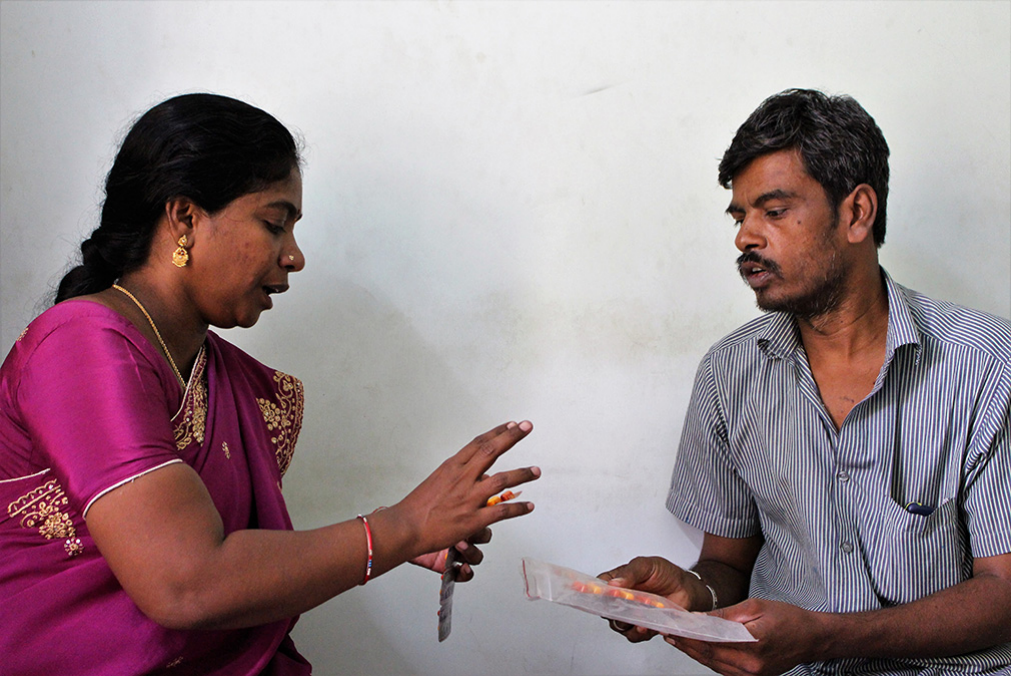
A staff member at an EQUIP center supports a patient to improve TB treatment literacy and adherence. © Jasper Hamann

The project demonstrated that effective participation of the private sector in TB control efforts in India is possible and can yield significant benefits to private providers and their patients as well as the public sector by encouraging appropriate TB diagnostic and treatment behaviors. Through EQUIP, DR-TB cases were identified quickly using state-of-the-art diagnostics, and they promptly received appropriate treatment with ongoing support for treatment adherence. Coordination through EQUIP as the interface agency between diverse private and public stakeholders and patients was critical to success. Key recommendations going forward are summarized in Box 2.

BOX 2 **Recommendations to Effectively Engage the Private Sector in TB Care**

- Ongoing outreach to private providers on a one-to-one basis and through other channels is necessary to maintain their interest in TB activities, given their many competing priorities. To maintain their motivation, providers' efforts to diagnose and notify TB cases to RNTCP and to prescribe appropriate, low-cost regimens to their patients should be recognized. The updated guidance from RNTCP allowing for daily regimens should be widely publicized, and providers should be encouraged to access RNTCP daily regimen drugs to treat their patients. These are labor-intensive activities that must be considered when planning private-sector initiatives.
- Private-sector engagement continues to require some sort of “interface” agency to play the coordination role between RNTCP, individual providers, and patients. This will likely continue to be the case until processes are streamlined, notification becomes mandatory, and quality services are widely available and affordable. The role and scope of the interface agency should be recognized and integrated within government reimbursement schemes.
- Continue to provide free or low-cost access to cartridge-based nucleic acid amplification tests (CB-NAAT), such as GeneXpert, as the initial diagnostic for private patients with TB symptoms. Make CB-NAAT available in private facilities, as Project EQUIP did. Encourage private providers to refer more of their symptomatic patients for CB-NAAT testing. Report back to them on the overall yield of their referrals and discuss why referring additional patients is warranted.
- Support for notification of TB cases through Nikshay is necessary to increase the proportion of notifications of private-sector patients. Although the online system allows access by private providers, most do not take the time to complete the forms, particularly the smaller clinics with few support staff. A more simplified process will be required to engage the private sector in the notification process.
- Providing patient- and provider-centered services is an essential component of any private-sector engagement model. Advocacy for expansion of the EQUIP treatment model can help maintain excellent treatment success rates.
- All private provider types can contribute substantially to increasing TB case notification and early DR-TB case detection and should be engaged in TB control efforts. Using a database capable of tracking referrals and TB cases diagnosed by individual provider can help target further efforts to engage the private sector by identifying high performers as well as areas for improvement.
- Reasons for the unexpectedly high yield of cases from referrals should be explored further to inform revisions to the approach that could increase the number of patients referred.
- A direct comparison of all successful private engagement models to combine the best practices of each could result in an optimized approach for private-sector engagement in India.

Effective participation of the private sector in TB control efforts in India is possible.

### High Level of Engagement of Private Providers

The level of participation by private providers (almost 50%) was much higher than anticipated (an estimated 10% to 15% based on previous REACH private-sector engagement work) and can be attributed to the benefits providers received from the project's services. First, we involved providers in the design of the model and individualized the approach to sensitization and follow-up. Second, we offered providers a comprehensive range of services: sputum collection and transport; free-of-charge GeneXpert testing with rapid turnaround times; and free-of-charge, quality daily treatment regimens for drug-sensitive TB while retaining their patients. Third, we provided reliable referral of DR-TB patients to public RNTCP clinics for second-line treatment.

While participation in the project was high, a much smaller subset of providers (the “super-referrers” who provided more than 30 referrals each) accounted for a high proportion of the patients referred. These super-referrers could be the focus of future efforts to continue private-sector engagement in the case that resources are limited. However, in order to identify this subgroup of providers, referrals and yields must be tracked by individual provider, requiring a substantial data collection and analysis effort.

### High Yield of Cases Among Patients Referred for GeneXpert

The yield of TB cases among people referred for GeneXpert testing was extremely high, with 47% of all referrals resulting in a confirmed TB diagnosis. In general, one would expect that about 10% of people with symptoms would have a positive TB diagnosis in a high-burden setting. True interpretation of these data is not possible without gathering further information about provider referral habits, which will continue in the ongoing phase of the work. Several possible explanations for the high yield exist. Providers may have concentrated on referring only those for whom they had a very high index of suspicion for TB based on chest x-ray or clinical presentation rather than referring all those with symptoms that could have been related to TB. In addition, they may have prescribed a course of general antibiotics first to rule out other causes of illness before referring for TB evaluation. They may also have preferentially referred patients with clinically diagnosed TB in whom they wanted to rule out drug resistance. Because this was the first year of engagement for many of the providers, it also may simply take more time to establish a trusting relationship that will encourage them to refer more of their clients for diagnosis. Although still high, the yield (proportion of cases diagnosed over the number referred) did decrease markedly over time among all provider groups, as shown in Table 3. This may be related to an increase in trust and therefore an increase in willingness to refer patients, as well as a gain in knowledge about who, how, and where to refer patients for testing.

### Private Providers' Contribution to Increasing Both TB and DR-TB Case Notifications

Prior to the project, most private-sector TB patients were not notified to the RNTCP and did not have a treatment outcome recorded. Mandatory notification of TB was instituted in 2012, but few private providers complied with the requirement because they were unaware; did not have appropriate forms and contacts to perform the task; refused due to concerns about patient confidentiality; or did not allocate time to do so. These barriers accounted for low notifications in previous years—for instance, total private-sector notifications for all of Chennai accounted for only 524 TB cases in 2015.

During the period October 2015 through June 2017, in total 12,171 TB patients were notified from Central and South Chennai to the RNTCP. In addition to the 1,232 patients diagnosed through EQUIP, private providers also requested EQUIP to notify 36 patients they had diagnosed and managed themselves. These 1,268 patients accounted for approximately 10% of all TB notifications to the RNTCP from these districts in the same period.

Twenty-six DR-TB cases were notified through EQUIP during the project period. In comparison, a total of 160 DR-TB cases were notified with the RNTCP in all of Chennai in 2016. While a direct comparison with overall DR-TB notification is not possible because of the differing time frames and geographies of RNTCP and EQUIP data, the contribution of EQUIP to DR-TB diagnosis and notification is nevertheless considerable, estimated to be similar to the contribution to drug-sensitive notifications at approximately 10% of DR-TB cases notified.

### Treatment at EQUIP Centers Preferred

Most (74%) of the 1,167 drug-sensitive TB patients who started treatment were treated through the private EQUIP treatment centers, which expanded from 4 centers at the beginning of the project to 13 centers at project conclusion. Of the patients treated at the EQUIP centers, 80% received daily treatment. Only 9% of drug-sensitive TB patients diagnosed through the EQUIP center were treated at an RNTCP center, usually when this center was more conveniently located for the patients. Approximately 16% were treated with anti-TB regimens under private prescription without treatment support from EQUIP, and only 0.25% were treated using non-standard traditional medicine.

### Improved Diagnostic and Treatment Practices and Good Treatment Outcomes

During 7 quarters of active project operations, the private sector referred 2,621 patients and diagnosed 1,232 TB cases in the Central and South districts of Chennai, approximately 10% of the total cases notified in these districts during the period. Without the project, it is likely that few of these cases would have been notified to the RNTCP and would have remained among the “missing” cases instead. Of the cases diagnosed in the private sector, 694 (56%) were bacteriologically confirmed using GeneXpert, a technology that was rarely used in the private sector in Chennai prior to the project. While this is a great improvement in bacteriological confirmation of TB in the private sector, there is room for further improvement. Private providers continue to rely heavily on chest x-ray for diagnosis and have few means of confirming extrapulmonary TB, which continues to be diagnosed primarily by clinical judgment.

The private-sector model contributed about 10% of the total TB cases notified in the project districts.

The project demonstrated high levels of private provider adherence to standardized, quality-assured treatment regimens, with more than 82% of patients prescribed a standard daily or intermittent regimen with quality-assured drugs. Perhaps even more important, their willingness to access treatment adherence support through EQUIP or RNTCP to ensure better outcomes for their patients produced excellent results. At the time of writing, 89% of patients who were eligible to complete treatment had done so successfully, approaching global and national targets for treatment success. Prior to the project, most private-sector patients did not have a treatment outcome recorded at all. Our result compares favorably with the 84% treatment success (42% cure and 43% treatment completed) for the full RNTCP cohort (2016 Q1–Q3 patient cohort; data obtained from RNTCP).

### Limitations

Notwithstanding the success of our approach in facilitating quality diagnosis, treatment, care, and notification for patients seeking care in the private sector in Chennai, some limitations are to be mentioned. The main limitation is the absence of key baseline data to compare against the data collected during the project because these data did not exist. The value of this project also lies in beginning the process of actually collecting data to quantify the potential contribution of the private sector in detecting TB and DR-TB. Without having had the opportunity to collect such baseline data ourselves through the private providers, we have no comparator related to the number of private providers engaged and the number of referrals per provider. Also, we do not know the proportion of all patients and eligible patients referred for diagnosis, as providers were unwilling to provide such data. The high proportion of patients diagnosed with TB indicates selective referral of patients, as discussed earlier. Whether it implies we missed a substantial proportion of TB diagnoses among the client population of the providers engaged will require follow-up research. The proportion of bacteriological confirmations did go down during the project period (78% in the fourth quarter of 2015 to 40% in the second quarter of 2017; data not shown), which indicates that with time private providers may be referring an increasing proportion of presumptive TB patients for bacteriological testing.

## CONCLUSION

The 13 EQUIP centers, now renamed Nakshatra (“Star”) centers, are a key element of the TB Free Chennai Initiative, led by the Corporation of Chennai, which plans to expand the REACH-led Nakshatra centers to a total number of 36 in Chennai. The TB Free Chennai Initiative will receive funding from USAID (to local government) and the Stop TB Partnership (to REACH). Within the TB Free Chennai Initiative, GeneXpert testing will continue to be available through the previous EQUIP-networked private hospital using the voucher system. As under EQUIP, this work will still be financed through external funding (USAID). Other mechanisms and new diagnostic tools are needed to make diagnostic testing more affordable for the private sector.^18^ In addition, domestic funding support will be required to ensure sustainability of the model, like all public-private models in India.

Although the private-sector engagement model has been adopted for scale-up, sustainability remains a concern.

REACH as the interface NGO takes care of sputum collection and transport, quick results delivery, and treatment adherence support to make the services as patient-friendly as possible. The Nikshay online case reporting platform has improved accessibility to reporting for private providers but remains time-consuming and will require the support of an interface NGO unless the amount of data required is reduced. The coming years should be used to develop mechanisms for government funding to support this type of qualified interface NGO. Given that demand is much greater than existing public health services can cover, these public-private interface models are important to extend quality diagnostic and treatment services to the majority of people with TB in India, many of whom prefer to seek care in the private sector.

## Notes

**Peer Reviewed**

**Funding:** United Way Worldwide grant, made possible by the Eli Lilly and Company Foundation.
